# Epiretinal membrane development after Ex-Press glaucoma filtration device implant: 2-year results of a case control study

**DOI:** 10.1007/s10792-024-02958-5

**Published:** 2024-02-17

**Authors:** Francesco Sartini, Martina Menchini, Alessandro Palma, Giamberto Casini, Michele Figus

**Affiliations:** Ophthalmology, Department of Surgical, Medical, Molecular Pathology and Critical Care Medicine, University of Pisa, Via Savi, 10, 56126 Pisa, Italy

**Keywords:** Ex-Press glaucoma filtration device, Epiretinal membrane, Glaucoma surgery, Cellophane macular reflex, Primary open-angle glaucoma

## Abstract

**Background:**

The most common retinal complications after glaucoma surgery are choroidal detachment, hypotony maculopathy, malignant glaucoma, vitreous hemorrhage, endophthalmitis and retinal detachment. However, if glaucoma surgery is a risk factor for the ERM development needs to be clarified. This study aims to assess the incidence of epiretinal membrane (ERM) in 2 years of follow-up in patients with primary open-angle glaucoma (POAG) treated with Ex-Press shunt implant.

**Methods:**

A prospective, consecutive, single-center, case–control study. We enrolled patients affected by POAG and scheduled for Ex-Press device implant with or without concomitant cataract surgery. The control group was the contralateral eyes which continues anti-glaucomatous eyedrops. Complete ophthalmologic evaluation and spectral-domain optical coherence tomography were performed before surgery, at 6 months and 24 months of follow-up.

**Results:**

Eighty-two eyes of 41 consecutive patients, 18 males and 23 females with a mean age of 70, 29 ± 8,45, were analyzed at 24 months. 39.1% of eyes developed ERM: 29.3% were cellophane macular reflex (CMR) and 9.8% were pre-macular fibrosis (PMF). In the control group, 19.5% of eyes developed ERM: 17.1% were CMR and 2.4% were PMF. No statistically significant difference was reported (*p* = 0.121) between treated and control group. ERM development did not affect significantly the central foveal thickness (260.13 ± 35.01 μm at baseline, 265.03 ± 34.90 μm at 6 months and 275.18 ± 33.31 μm at 24 months) and macular volume (7.75 ± 0.43 mm^3^ at baseline, 7.77 ± 0.48 mm^3^ at 6 months and 7.77 ± 0.46 mm^3^ at 24 months), remained comparable to reported average measures in healthy individuals during the follow-up. Concomitant cataract surgery did not increase the ERM incidence.

**Conclusion:**

Ex-Press implant may increase the ERM incidence regardless concomitant cataract surgery, accelerating or inducing a posterior vitreous detachment, such as other ocular surgical procedure. Nevertheless, the vast majority of ERM are CMR, not affecting the macular profile.

## Background

Epiretinal membrane (ERM) seems due to proliferation of glial cells, astrocytes, fibrocytes and myofibroblasts after migration through defects in the internal limiting membrane [1]. Even if ERM is idiopathic in most cases, several ocular conditions have been reported as risk factor: diabetic retinopathy, ocular trauma, inflammatory disease and ocular surgery [2]. The most common retinal complications after glaucoma surgery are choroidal detachment, hemorrhagic choroidal detachment, hypotony maculopathy, malignant glaucoma, vitreous hemorrhage, endophthalmitis and retinal detachment [3]. However, if glaucoma surgery is a risk factor for the ERM development need to be clarified. In a previous study we evaluated the frequency of ERM development after Ex-Press (Alcon Laboratories, Fort Worth, TX, USA) shunt implantation in patients affected by primary open-angle glaucoma (POAG) after six months of follow-up [4]. We also assessed the role of concomitant cataract surgery and the impact of ERM on central foveal thickness (CFT) and macular volume (MV). In this study, we present our results after two years after Ex-Press implant in the same population.

## Methods

The study design and methods are detailed in our previous study [4], nevertheless, are also briefly summarized here.

Of the 54 patients enrolled at 6 months, 13 were lost at follow-up; therefore, 41 patients with POAG and scheduled for Ex-Press glaucoma filtration device implant with or without concomitant phacoemulsification were included in this prospective, consecutive, single-center, case–control study. We received approval by the local Institutional Review Board (CEAVNO, Comitato Etico Area Vasta Nordovest, register number: 16554-Casini). All the procedures were conducted according with the Declaration of Helsinki, and every patient signed an informed consent form. Surgical procedures were performed by the same surgeon in the eye clinic of Pisa University Hospital (Pisa, Italy), between October 2018 and October 2019.

We performed a complete ophthalmic evaluation including best-corrected visual acuity (BCVA), Goldmann applanation tonometry, gonioscopy, standard automated perimetry, biomicroscopy and optical coherence tomography (OCT) (Spectralis, Heidelberg Engineering, Heidelberg, Germany) at baseline, 6 and 24 months postoperatively.

The diagnosis of POAG was based on gonioscopy, optic disk imaging and visual fields defects on standard automated perimetry. Inclusion criteria were diagnosis of POAG, indication for surgical treatment, contralateral eye affected by POAG and treated with anti-glaucoma eyedrops.

The exclusion criteria were previous or subsequent ocular laser or surgical treatments except for cataract surgery at least 24 months before enrollment, preexisting retinal pathology (such as schisis, vascular retinal diseases, ERM, choroidal neovascularization and age-related macular degeneration), preexisting posterior vitreous detachment (PVD), ocular trauma and ocular inflammatory diseases.

ERM was classified in two types: (1) Cellophane macular reflex (CMR) defined as hyperreflective vitreomacular interface without foveal depression loss or alteration of the extrafoveal architecture; (2) pre-macular fibrosis *(*PMF) defined as OCT hyperreflective layer with foveal depression loss, with or without intraretinal fluid, and alteration of the extrafoveal architecture due to the ERM contraction [4].

**Fig. 1:**
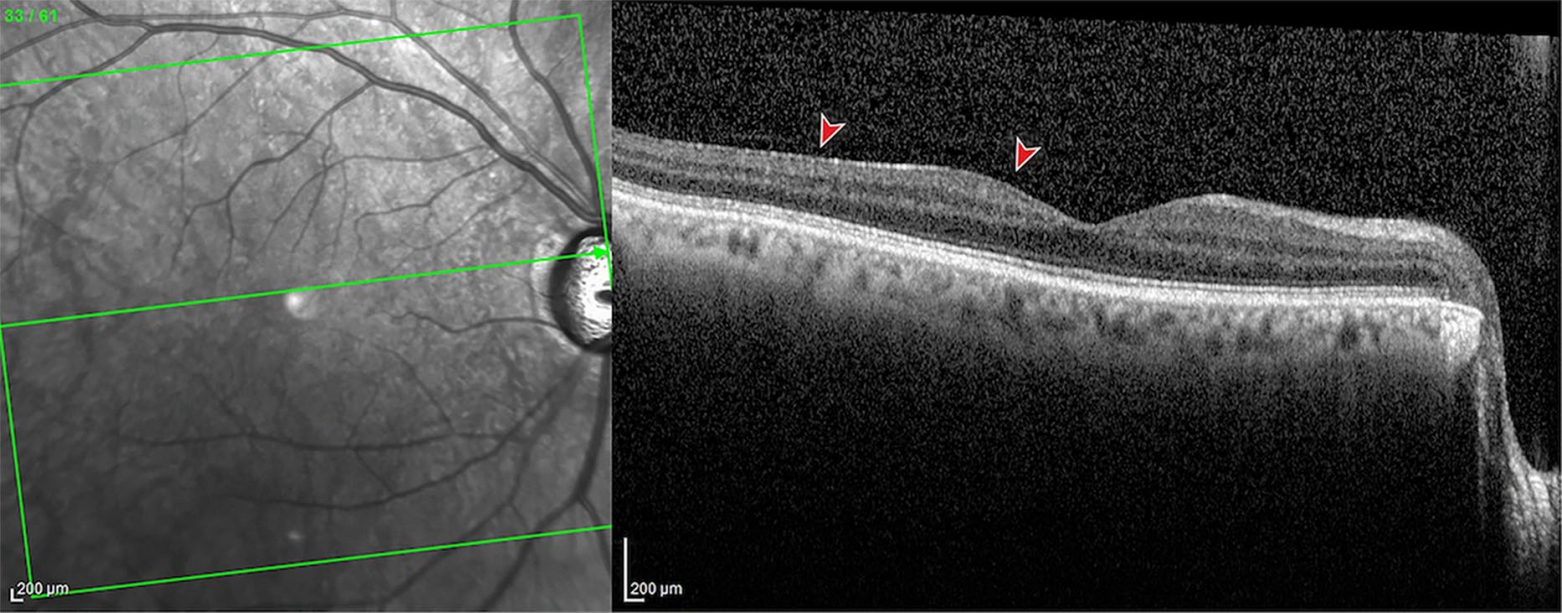
Optical coherence tomography of a cellophane macular reflex. An hyperreflective layer can be noted at the vitreomacular interface without foveal depression loss. Reproduced with permission from [4]

**Fig. 2:**
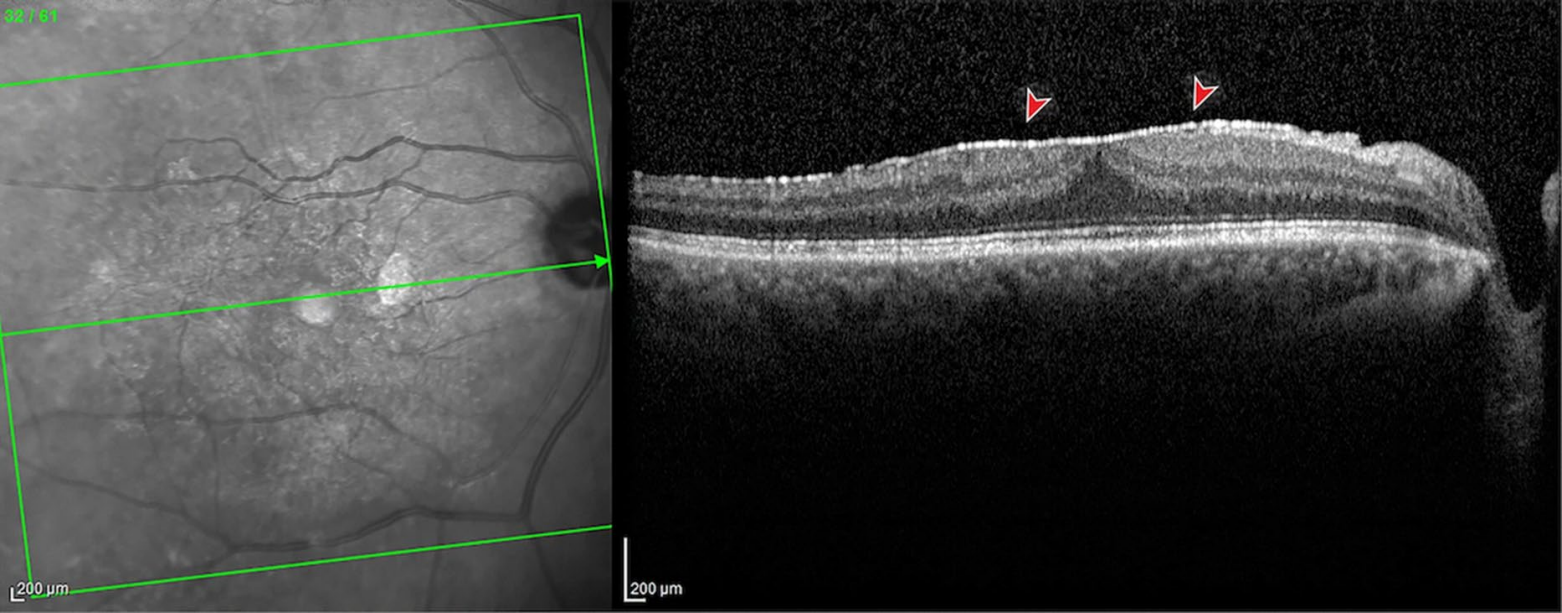
Optical coherence tomography of pre-macular fibrosis. An hyperreflective layer with foveal depression loss, due to the ERM contraction. Reproduced with permission from [4]

OCT was evaluated by to blinded experienced observers and, in case of discordance, the principal investigator reviewed and categorized the images (Figs. 1 and 2).

The occurrence of ERM was compared between treated eyes and the control group (contralateral eyes affected by glaucoma and receiving anti-glaucoma eyedrops) and between Ex-Press implant alone and Ex-press implant with concomitant cataract surgery. We calculated CFT and MV using the Early Treatment Diabetic Retinopathy Study (ETDRS) grid, comparing values between the above-mentioned groups. CFT was defined as the average thickness of the macula in the central 1-mm ETDR grid. MV was defined as the sum of all volumes of all nine subfields.

### Surgical procedures

The surgical procedure was described in detail in our previous article [4].

### Statistical analysis

Statistical analysis was performed using SPSS Statistics version 25 (IBM corporation, Armonk, NY, USA). Descriptive statistics was used to summarize mean values and standard deviations of all numerical data. Sample size was calculated using the effect size from the results of a previous similar study [1] and indicated that 54 subjects were required to detect a 25.7% difference in the incidence of ERM, with a power of 80% and a significance level of 0.05. Post hoc analysis indicated that this study had a power of 90% with an actual *α* of 0.02 to detect a 24% difference in the incidence of ERM between treated eyes and controls. The distribution of values was assessed using the Kolmogorov–Smirnov test and the Shapiro–Wilk test. For normally and not normally distributed continuous variables, the ANOVA test for repeated measures and the Kruskal–Wallis test were used to compare baseline with follow-up measures (6 months and 24 months). Then BONFERRONI post hoc test was performed if the previous test’s results were significant. *χ*2 test was used for categorical variables. *P* values < 0.05 were considered significant [4].

## Results

Eighty-two eyes of 41 consecutive patients, 18 males and 23 females with a mean age of 70,29 ± 8,45, were analyzed at 24 months. Average time of follow-up was 24,34 ± 1,29. Twenty-two patients were treated with Ex-Press implant and phacoemulsification (53.7%) and 19 with Ex-Press implant alone (46.3%).

At six months of follow-up in the treated group 12 cases (29.3%) of ERM were observed: 10 (24.9%) of CMR and 2 (4.9%) of PMF. In the control group, 8 eyes (19.5%) developed an ERM: 7 (17.1%) CMR and 1 (2.4%) PMF. No statistically significant difference was reported (*p* = 0.797). At 24 months in the treated group, 16 (39.1%) eyes developed ERM: 12 (29.3%) were CMR and 4 (9.8%) were PMF. In the control group, 8 eyes (19.5%) developed ERM: 7 (17.1%) were CMR and 1 (2.4%) was PMF. No statistically significant difference was reported (*p* = 0.121) between treated and control group.

Regarding the type of surgery, the ERM frequency did not statistically differ between eyes subjected to combined surgery (6 eyes) and eyes treated with the Ex-Press implant alone (6 eyes) (*p* = 0.286) at 6 months. In the combined surgery group, 4 CMR and 2 PMF were reported; in the Ex-Press implant alone, all the ERM were CMR (6). Similar results were obtained at 24 months of follow-up, 9 ERM were developed in the combined group (6 CMR and 3 PMF) and 7 in the Ex-Press implant alone (6 CMR and 1 PMF), with no statically significant difference (*p* = 0.662).

The CFT and the MV were compared between treated and control eyes at baseline, at 6-month and 24-month follow-up visits. No statistical difference was noted at each time point among groups.

Also, CFT and MV were compared between eyes with and without the ERM within treated group, and differences were not statistically significant.

Finally, no difference in mean CFT and MV was observed within treated eyes between simple and combined surgery (Table 1, 2 and 3).

**Table 1:** Demographic and preoperative features

Gender	Male	18 (43.9%)
	Female	23 (56.1%)
Mean age (y)	70, 29 ± 8,45	
Treatment	Ex-Press implant alone	22 (53.7%)
	Ex-Press implant and cataract	19 (46.3%)
IOP (mmHg)	Control group	12.5 ± 1.7
	Treated group	31.2 ± 7.9
Mean number of eyedrops	Control group	1.99 ± 0.99
	Treated group	2.85 ± 0.76
Mean MD	Control group	*− *9.67 ± 4.9
	Treated group	− 13.12 ± 6.8
Mean PSD	Control group	6.44 ± 3.7
	Treated group	10.35 ± 2.29
CFT baseline (μm)	Control group	250.47 ± 28.77
	Treated group	260.13 ± 35.01
MV baseline (mm^3^)	Control group	7.75 ± 0.43
	Treated group	7.84 ± 0.39

*IOP* Intraocular pressure, *MD* Mean deviation, *PSD* Pattern standard deviation, *CFT* Central foveal thickness, *MV* Macular volume, *SD* Standard deviation, *y* years

**Table 2:** Epiretinal membrane after Ex-Press glaucoma filtration device implant

	Treated				Control	
	Ex-Press (*n* = 22)	Phaco-Ex-press (*n* = 19)	Total (*n* = 41)	*p*	*n* = 41	*p*
ERM (%) at 6 months	6	6	12 (29.3)	0.286	8 (19.5)	0.797
CMR (%) at 6 months	6	4	10 (24.9)		1 (2.4)	
PMF (%) at 6 months	0	2	2 (4.9)			
ERM at 24 months	7	9	16 (39.1)	0.662	8 (19.5)	0.121
CMR (%) at 24 months	6	6	12 (29.3)		7 (17.1)	
PMF (%) at 24 months	1	3	4 (9.8)		1 (2.4)	

*CMR* Cellophane macular reflex, *PMF* Pre-macular fibrosis

**Table 3:** Epiretinal membrane after Ex-Press glaucoma filtration device implant

	Treated							Control	
	Total	Ex-Press	Phaco-Ex-Press	*p*^a^	ERM	No ERM	*p*^b^	Total	*p*^c^
CFT at 6 months	265.03 ± 34.90	262.50 ± 35.74	269.28 ± 36.18	0.520	260.92 ± 33.57	267.11 ± 39.13	0.188	258.61 ± 32.58	0.580
MV at 6 months	7.77 ± 0.48	7.64 ± 0.45	7.90 ± 0.46	0.096	7.82 ± 0.61	7.74 ± 0.40	0.503	7.84 ± 0.45	0.332
CFT at 24 months	275.18 ± 33.31	272.83 ± 31.90	278.89 ± 36.71	0.537	268.56 ± 31.14	282.45 ± 35.92	0.455	268.50 ± 27.65	0.239
MV at 24 months	7.77 ± 0.46	7.70 ± 0.49	7.84 ± 0.44	0.385	7.79 ± 0.60	7.76 ± 0.40	0.503	7.84 ± 0.46	0.351

*CFT* Central foveal thickness, *MV* Macular volume, *ERM* Epiretinal membrane

^a^Ex-Press versus Phaco-Ex-Press, ^b^ERM versus no ERM, ^C^treated versus control

Intraocular pressure (IOP) did not change during the study period in the control group (12.5 ± 1.7 mmHg at baseline, 12.7 ± 1.9 mmHg at 6 months and 13.8 ± 2.5 mmHg, respectively *p* = 0.583). Otherwise, IOP of the treated eyes significantly decreased from baseline to 6 months (respectively, 31.2 ± 7.9 mmHg and 11.8 ± 3.1 mmHg (*p* = 0.002)) and remained stable from 6 to 24 months (respectively, 11.8 ± 3.1 mmHg and 12.0 ± 3.4 mmHg (*p* = 0.754)). The mean number of anti-glaucomatous eyedrops of treated eyes decreased from 2.85 ± 0.76 to 0.13 ± 0.44 at 6 months (*p* = 0.008) and remained stable from 6 to 24 months (respectively, 0.13 ± 0.44 and 0.18 ± 0.72 (*p* = 0.898)).

Mean deviation (MD) and pattern standard deviation (PSD) were collected from all patients. No difference was observed between baseline and postoperative values of mean MD in the treated group (respectively, − 13.12 ± 6.8 and − 14.14 ± 5.16 and − 15.31 ± 4.5, respectively, *P* = 0.454). Also, the PSD did not change significantly in the treated group during follow-up (respectively, 10.35 ± 2.29 at baseline, 9.68 ± 5.73 at 6 months and 12.08 ± 2.67 at 24 months *p* = 0.784)).

In the control group, mean MD (− 9.67 ± 4.9 at baseline, − 9.02 ± 4.2 at 6 months and − 9.89 ± 4.9 at 24 months) and mean PSD (6.44 ± 3.7 at baseline, 7.6 ± 3.3 at 6 months and 8.03 ± 3.23 at 24 months) did not significantly change during the follow-up period (*p* = 0.370 and *p* = 0.606, respectively).

Visual acuity significantly increased postoperatively in eyes subjected to combined surgery, from 0.23 ± 0.11 logarithm of minimum angle of resolution (logMAR) to 0.08 ± 0.10 logMAR (*p* = 0.001) and remained stable at 24 months (0.10 ± 0.12 logMAR, *p* = 0.912). Visual acuity did not significantly change in the eyes not subjected to cataract surgery (0.18 ± 0.28 logMAR at baseline, 0.16 ± 0.32 logMAR at 6 months and 0.21 ± 0.33 logMAR at 24 months, *p* = 0.879).

## Discussion

ERM incidence in our control group (19.5%) at 24 months, this value is comparable to data available in the literature (2.2–26.1%), bearing in mind that patients’ mean age was 70, 29 ± 8,45, corresponding to the peak of ERM incidence reported by the Blue Mountains Eye Study between 74 and 85 years of age, with a stable prevalence in those older than 85 years [5, 6].

Ocular surgery plays an important role in ERM development. Patients undergone to cataract surgery reported an ERM incidence of 16.8% and following pars plana vitrectomy for rhegmatogenous retinal detachment ERM incidence ranged from 4.4 to 12.8% [7–9]. Nevertheless, literature about ERM incidence after glaucoma surgery is poor.

Vieria et al. reported an ERM incidence after trabeculectomy of 56%, of whom 9/50 eyes (18%) with PMF and 19/50 eyes (38%) with CMR. The mean follow-up time after surgery was 27.8 months [1].

In this study, the ERM incidence was 39.1% at 24 months, 12 eyes (29.3%) developed a CMR and 4 eyes (9.8%) a PMF. At 6 months after the surgery, ERM incidence was 29.3%; in particular, 12 (29.2%) eyes developed CMR and 2 (4.9%) PMF.

It is interestingly to note that ERM frequency increased, but CMR remained the most frequent ERM type with no alteration of macular profile. In the PMF cases, patients did not experienced vision loss or metamorphopsia at both time points during the follow-up.

Additional cataract surgery did not increase the ERM incidence after Ex-Press implant, because no significant difference was reported between ERM frequency in the combined surgery group and Ex-Press implant alone at each time points.

CFT and MV did not change significantly during the follow-up in the treated group. Also, no difference in terms of CFT and MV has been noted in the subgroup analysis between combined surgery and Ex-Press implant alone. Interestingly, CFT and MV remained comparable to reported average measures in healthy individuals ranging from 255.4 to 271.4 μm and from 6.76 to 8.53 mm3, respectively [10].

Furthermore, CFT and MV did not significantly increase between eyes with and without the ERM. This point confirms that most of ERM was CMR not altering the macular profile, and the 4 cases of PMF reported no visual loss or other symptoms.

Moreover, OCT is more sensible to detect ERM compared to biomicroscopy or fundus photography, increasing the incidence of ERM in patients where diagnosis is made with OCT. Therefore, ocular surgery is surely a risk factor developing ERM, but its impact should be overestimated, especially comparing the ERM frequency after surgery diagnosed with OCT, versus ERM incidence in healthy population, usually diagnosed with less sensitive methods.

In particular, a recent cross-sectional study on the same population of the Blue Mountains Eye Study reported ERM incidence of 21.4% using OCT for diagnosis, compared to the previous reported value of 7%, when biomicroscopy and stereo fundus photography had been used to make the diagnosis [5] [7]. Therefore, photographic diagnosis may underestimate ERM incidence by two- to threefold compared with OCT [5].

Furthermore, ERM was associated strongly with increasing age, a relationship that also has been reported in most other studies of ERM [11].This is likely related to the onset of PVD [12]. Cataract surgery is associated with ERM development, as already reported other studies, supporting the role of PVD in ERM development [6, 13, 14].

Nevertheless, the exact mechanism that leads to ERM formation is still debated, because the myofibroblastic pre-retinal cells are thought to transdifferentiate from glial and retinal pigment epithelial cells that reach the retinal surface via defects in the internal limiting membrane (ILM) or from the vitreous cavity [2].

Therefore, Ex-Press implant alone or combined with cataract surgery increases the incidence of ERM (39.1%), but less then trabeculectomy (56%) and more than cataract surgery alone (16.8%) [1, 7].

Therefore, we support the role of PVD in increasing ERM incidence, but Ex-Press implant surgery is less invasive compared to trabeculectomy, as demonstrated a minimal inflammation and scarring reactions after Ex-Press implant in rabbits [15]. The increased levels of postoperative inflammation and the rapid variation of IOP that occur after glaucoma surgery may induce or accelerate the PVD.

Nevertheless, Ex-Press implant procedure is less invasive compared to trabeculectomy, because iridectomy is not necessarily performed, maybe justifying the reduced ERM incidence in our study compared to data reported by Viera et al. [1].

Moreover, any role of postoperative hypotony was excluded because early postoperative IOP did not show a statistical difference between eyes that developed ERM and eyes without.

Also, the role of retinal break in ERM development after Ex-Press implant can be excluded, because during the follow-up period no treated eye experienced a retinal break or a retinal detachment.

A limitation of our study is the loss of 13 patients during the follow-up compared to our previous study population. Nevertheless, the calculation of sample size confirmed that a minimum of 38 patients was required to detect a 25.7% difference in the incidence of ERM, with a power of 80% and a significance level of 0.05.

Another limitation is the subjective evaluation of OCT images; however, each masked researcher analyzed all raw images of the macular area to detect any artifact, before evaluating the macular profile. In case of discordance between the masked researchers, the principal investigator reviewed and categorized the images.

## Conclusions

Ex-Press implant confirms to be a comparable to trabeculectomy in terms of IOP reductions at 24 months, reducing the mean number of anti-glaucomatous eyedrops. As other surgical procedure, Ex-Press implant may increase the ERM incidence regardless of concomitant cataract surgery, accelerating or inducing a PVD.

Nevertheless, the vast majority of ERM is CMR, not affecting the macular profile, and consequently with a really limited impact on clinical practice.

## Data Availability

The datasets used and/or analyzed during the current study are available from the corresponding author on reasonable request.

## Abbreviations

BCVA: Best-corrected visual acuity
CFT: Central foveal thickness
CRM: Cellophane macular reflex
ERM: Epiretinal membrane
MV: Macular volume
OCT: Optical coherence tomography
PMF: Pre-macular fibrosis
POAG: Primary open-angle glaucoma
PVD: Posterior vitreous detachment
